# Diagnostic accuracy of the Hunger Vital Sign screener for food insecurity in an obstetric population

**DOI:** 10.1002/pmf2.70238

**Published:** 2026-01-24

**Authors:** Tazim Merchant, Samantha McLean, Meghan Ludke, Ying Cheung, Priyanka Mathur, Sydney Raucher, Charlotte Niznik, Joe Feinglass, Lynn M. Yee

**Affiliations:** ^1^ Department of Obstetrics and Gynecology Northwestern University Feinberg School of Medicine Chicago Illinois USA; ^2^ Department of Obstetrics and Gynecology Mayo Clinic Rochester Minnesota USA; ^3^ Division of Biostatistics Department of Preventive Medicine Northwestern University Feinberg School of Medicine Chicago Illinois USA; ^4^ Department of Obstetrics and Gynecology Brigham and Women's Hospital and Massachusetts General Hospital Boston Massachusetts USA; ^5^ Division of General Internal Medicine, Department of Medicine Northwestern Feinberg School of Medicine Chicago Illinois USA

**Keywords:** food access, food insecurity, hunger vital sign (HVS), social determinant of health

## Abstract

**Introduction:**

The Hunger Vital Sign (HVS) is a two‐item, validated food insecurity screener recommended by the American Academy of Pediatrics. Given its brevity, HVS could be useful in assessing food insecurity in pregnant populations, for whom high‐quality nutrition and food access is essential. However, the HVS has not been evaluated in obstetric settings. Thus, we aimed to evaluate the performance of the HVS compared to the gold standard, the 18‐item US Household Food Security Survey Module (HFSSM) among low‐income, urban pregnant people.

**Methods:**

We conducted a prospective study at a multi‐hospital tertiary health system (September 2023 to April 2024) in the United States. English‐ or Spanish‐speaking individuals from zip codes in which ≥10% of households were under the federal poverty line or who had publicly funded antenatal care completed a 20‐min self‐administered survey, which included the HFSSM and HVS. Food insecurity was defined as marginal, low, or very low food insecurity on the HFSSM; the prevalence of food insecurity was assessed and evaluated by participant characteristics. A positive screen on the HVS was defined as a “often true” or “sometimes true” to either item. Descriptive statistics were performed to characterize the performance of the HVS compared to the HFSSM.

**Results:**

Of 713 potentially eligible participants based on medical record review, 192 (27%) completed an eligibility screener and were found to be eligible. Of these, 171 (89%) completed the survey. Nearly half (41%) of participants (*n* = 70; of whom 74% received publicly funded care) screened positive for food insecurity on the HFSSM. HVS sensitivity was 83%, while specificity was 96%. The positive predictive value of the HVS was 94% and negative predictive value was 89%.

**Conclusion:**

The two‐item HVS had relatively high accuracy for detecting food insecurity in this low‐income, urban obstetric population. Incorporating this brief screener into prenatal care may enable efficient, clinically useful food insecurity detection and intervention to address this need.

## 1 INTRODUCTION

Food insecurity, defined as “being uncertain of having, or unable to acquire, enough food to meet the needs of all family members at times during the year,” is a critical social determinant of health. This public health challenge disproportionately affects pregnant individuals, for whom consistent access to sufficient quality and quantity of nutritious food is essential to promote perinatal health and reduce adverse pregnancy outcomes [1]. Despite these needs, fewer than 30% of pregnant individuals meet guideline‐adherent dietary intake, often due to accessibility challenges [2]. Additionally, food insecurity disproportionately affects individuals who identify as women, with disparities further evident across race, ethnicity, and socioeconomic status [2].

Given its association with adverse perinatal outcomes, including iron deficiency anemia, inappropriate gestational weight gain or loss, fetal growth issues, perinatal mental health conditions, and other obstetric complications, identifying and addressing food insecurity in pregnancy is of clinical and public health importance [3]. To effectively support populations with food insecurity, however, obstetricians must have access to an efficient, validated screening tool to promote the detection and intervention of food insecurity.

Various tools exist to screen for food insecurity across different patient populations. The 18‐item US Household Food Security Survey Module (HFSSM) is considered the gold standard; however, its length and complex scoring system limit its practicality in clinical settings [4, 5, 6]. The Hunger Vital Sign (HVS), a two‐item questionnaire developed by Hager et al. in collaboration with Children's HealthWatch, has been validated in pediatric and adult clinical settings [4]. This easy‐to‐administer tool recently demonstrated a high sensitivity of 94% in a study involving adults from emergency departments, compared to the HFSSM gold standard [6]. However, there are no studies of the HVS as a screening modality for food insecurity in pregnant populations. Given the unique nutritional, social, and health care needs of this group, the brief, two‐item tool could be a valuable prenatal care tool. This study's primary objective was therefore to assess the performance of the HVS compared to the HFSSM as a screening tool for food insecurity in a low‐income, urban, pregnant population.

## 2 METHODS

This prospective, survey‐based study was conducted at a multicenter tertiary health system in the greater Chicago, Illinois, region between September 2023 and April 2024. Potential participants were identified through reports from the institution's data warehouse, a repository containing information from electronic medical records (EMRs) and administrative systems. All patients receiving prenatal care at the institution's ambulatory practices that used this health system's EMR for prenatal care were screened for potential eligibility by the data warehouse automated processes.

Eligibility criteria included English‐ or Spanish‐speaking patients who were at or above a gestational age of 8 weeks, who either received publicly funded antenatal care or resided in zip codes where ≥10% of households have incomes below the federal poverty line, and who intended to deliver at a hospital in this health system. Exclusion criteria included a non‐viable pregnancy or intent to transfer to care outside institutions. Potentially eligible patients were contacted by trained research assistants through the EMR's patient portal using a standardized script. Interested potential participants followed a unique Research Electronic Data Capture (REDCap) link to an electronic screening questionnaire to confirm eligibility. If a patient met the eligibility criteria, informed consent was obtained electronically. Following consent, participants completed a self‐administered 20‐min survey, which included both the HFSSM and HVS measures, as well as questionnaires collecting demographic information including racial and ethnic identity, country of birth, relationship status, education level, occupation, and housing status. Participants received a $20 gift card as an incentive for completing the survey.

For both the HFSSM and HVS, a 12‐month recall period was used. Food insecurity was defined as marginal, low, or very low food security on the 18‐item HFSSM. The HVS is adapted from two items from the 18‐item HFSSM and identifies households at risk for food insecurity if they respond “often true” or “sometimes true” to either of the two statements [6]. The two statements include: “Within the past 12 months we worried whether our food would run out before we got money to buy more” and “Within the past 12 months the food we bought just didn't last and we didn't have money to get more.” According to the established criteria, an affirmative response to either question on the HVS indicated a positive screen for food insecurity [6].

Descriptive statistics were used to assess sociodemographic factors of the patient population in relation to the prevalence of food insecurity identified by the HFSSM. Sensitivity, specificity, positive predictive value, and negative predictive value were calculated for the HVS compared to the HFSSM. The primary outcome of the study was the diagnostic accuracy of the HVS in relation to the gold‐standard HFSSM based on these descriptive statistics.

As a secondary analysis, we assessed the sensitivity and specificity of the HVS as a tool for capturing different levels of food insecurity on the HFSSM including marginal, low, and very low food insecurity. We also calculated the sensitivity, specificity, positive and negative predictive values for at least low food security (i.e., low or very low food security vs. high or marginal) and for very low food security (i.e., very low vs. high, marginal, or low).

This study was approved by the Northwestern University Institutional Review Board and all participants provided written informed consent.

## 3 RESULTS

Of 713 potentially eligible participants who were contacted by trained research assistants, 192 (27%) were identified as eligible participants, and 171 (89%) consented to participation and completed the survey. Of these, 47% of the participants identified as Hispanic or non‐Hispanic Black. Approximately 40% of the participants reported a household annual income less than $50,000 and a little over a third of respondents (39%) owned homes. A majority (57%) of respondents were enrolled in a Medicaid for antenatal care. Participants with food insecurity were more likely to have Medicaid insurance, identify as non‐Hispanic Black, and have a lower income than those without food insecurity; they were also more likely to have a substance use disorder, have higher parity, and have higher preconception weight. Individuals with food insecurity were less likely to be married (Table 1).

**TABLE 1:** Demographics and clinical characteristics associated with food insecurity during pregnancy.

	Overall *N* = 171	No food insecurity *N* = 101	Food insecurity *N* = 70	*p* value
Insurance—Medicaid	97 (57%)	45 (45%)	52 (74%)	<0.001
Marital status				
Married	78 (46%)	62 (61%)	16 (23%)	<0.001
Living with partner	52 (30%)	21 (21%)	31 (44%)	
Other	41 (24%)	18 (18)	23 (33%)	
Homeowner	67 (39%)	49 (49%)	18 (26%)	0.003
Race/ethnicity				0.015
Non‐Hispanic White	69 (41%)	46 (46%)	23 (33%)	
Non‐Hispanic Black	33 (19%)	14 (14%)	19 (28%)	
Hispanic, any race	48 (28%)	26 (26%)	22 (31%)	
Asian	8 (4.7%)	8 (7.9%)	0 (0%)	
Other/multi‐racial	13 (7.6%)	7 (6.9%)	6 (8.7%)	
Education				0.005
Less than college	48 (28%)	21 (21%)	27 (40%)	
Associates degree/some college/college degree	78 (46%)	46 (46%)	32 (47%)	
Graduate degree or greater	44 (26%)	34 (34%)	10 (14%)	
Salaries				<0.001
Under $10,000	14 (8.2%)	4 (4.0%)	10 (14%)	
$10,000–25,000	17 (9.9%)	6 (5.9%)	11 (16%)	
$25,000–50,000	38 (22%)	14 (14%)	24 (35%)	
$50,001–100,000	42 (25%)	26 (26%)	16 (23%)	
$100,001–200,000	25 (15%)	23 (23%)	2 (2.9%)	
Over $200,000	17 (10%)	17 (17%)	0 (0%)	
Chronic hypertension	19 (11%)	5 (5.0%)	14 (20%)	0.002
Substance use disorder before or during pregnancy	9 (5.3%)	1 (1.0%)	8 (12%)	0.003
Parity (mean, SD)	1.2 (1.5)	1.0 (1.3)	1.5 (1.8)	0.047
Preconception weight (mean, SD)	79 (20.9)	75 (17.5)	84 (24.4)	0.04

*Note*: Data displayed as *N*(%) or mean (SD).

In this cohort, 41% of all participants (*n* = 70) screened positive for food insecurity on the HFSSM. Of those who screened positive, 74% had publicly funded antenatal care. Over one third (36%, *N* = 62) screened positive for food insecurity on the HVS (Table 2); 77% of these participants had publicly funded antenatal care.

**TABLE 2:** Hunger Vital Sign result based on the severity of food insecurity on Household Food Security Survey Module.

	HFSSM			
	High food security	Marginal food security	Low food security	Very low food security
	*N* = 101	*N* = 24	*N* = 26	*N* = 20
HVS				
Positive	4	13	25	20
Negative	97	11	1	0

*Note*: Data displayed as *N*.

Abbreviations: HFSSM, household food security survey module; HVS, hunger vital sign.

Overall HVS sensitivity for food insecurity was 83%, while specificity was 96%. The positive predictive value of the HVS was 94% and the negative predictive value was 89%.

On secondary analysis (Table 3), HVS was most sensitive for at least low food security (inclusive of very low) and very low food security, with sensitivity of 98% and 100%, respectively. The positive and negative predictive values of the HVS for low or very low food security (vs. high or marginal food security) were 73% and 99%, respectively. Similarly, the positive and negative predictive values of the HVS for very low food security (vs. high, marginal, or low food security) were 32% and 100%, respectively, in this small population. Finally, HVS was least sensitive for the category of marginal food security (excluding very low or low food security), with a sensitivity of 54%.

**TABLE 3:** Test characteristics of the Hunger Vital Sign for detecting levels of food insecurity versus the HFSSM.

	Marginal, low, or very low food security (*n* = 70)^a^	Low or very low food security (*n* = 46)^b^	Very low food security (*n* = 20)^c^
Sensitivity	83%	98%	100%
Specificity	96%	86%	72%
Positive predictive value	94%	73%	32%
Negative predictive value	89%	99%	100%

^a^ Compared with high food security.

^b^ Compared with high or marginal food security.

^c^ Compared with high, marginal, or low food security.

## 4 DISCUSSION

This study assessed the diagnostic accuracy of the two‐item HVS in detecting food insecurity compared to the 18‐item gold standard, the US HFSSM. In this largely urban population, our analysis demonstrated that HVS exhibited high sensitivity and specificity in detecting food insecurity within a low‐income antenatal setting. The positive and negative predictive values for food insecurity in this population were also high. The HVS's diagnostic accuracy, comparable to the gold‐standard HFSSM, suggests that it may be a useful tool to rapidly identify patients in need of additional resources to address food insecurity during prenatal care visits.

Additionally, the HVS has a high sensitivity for identifying individuals experiencing very low and low food security, underscoring its ability to detect those who may be at most need for critical food access–related resources. Given that food insecurity is correlated with other adverse social determinants of health in non‐pregnant and pregnant populations [1], and is associated with suboptimal health during pregnancy, patients who screen positive for food insecurity are likely at higher risk for both clinical morbidity and other social needs [2, 7]. This tool may therefore be valuable to identify those who may benefit from additional social needs screening and resources. This study's findings also suggest that if the clinical or public health goal is to detect marginal food security, the gold‐standard HFSSM may be more appropriate. Future work may be required to better understand how to identify this potentially vulnerable but underrecognized population with marginal food security, as these individuals may be particularly sensitive to unanticipated changes in resources or benefits.

Future research can also assess patient and clinician preferences regarding this screening tool, with focus on when and how to best screen, given the sensitivity of this issue and importance of resource provision. Our study was designed to specifically assess performance of the HVS in a pregnant population, and therefore future work is needed to understand implementation of this potential screening tool in a clinical context, its relationship to clinical outcomes, its value in assessing food insecurity among sub‐populations of pregnant people (such as those with diabetes or obesity), and its association with other social determinants of health. Hager and colleagues, who validated the HVS in a pediatric population utilizing data from over 30,000 caregivers in hospital‐based, primarily urban settings across the country, additionally reported whether households identified as food insecure on HVS but food secure on the HFSSM had statistically significant differences in outcomes such as hospitalization or depression. The authors found that caregivers in food‐insecure households were more likely to have a positive depression screen, and children in these households were 1.11 times as likely to have hospitalizations during their lifetime [4]. Similarly, Baer and colleagues, in addition to assessing diagnostic accuracy, also reported on how HVS score was significantly associated with the presence of other social determinants of health outcomes [4, 5]. Future research on this tool in pregnancy should additionally assess the HVS's ability to screen for such outcomes in relation to the HFSSM in order to further validate its use in prenatal care settings.

This study's strengths include addressing a gap in the literature regarding antenatal food insecurity and providing evidence to validate the usage of HVS in obstetric populations. The study population reflected a low‐income and diverse population, with nearly 50% of individuals identifying as Hispanic or non‐Hispanic Black and all participants meeting individual or neighborhood criteria for low income. This study's primary limitation is its sample size and limitation to a single, urban geographic region. Future work could validate the use of HVS in a larger population of pregnant people, assessing both the diagnostic accuracy and feasibility of utilizing this screener in multiple practice settings to ascertain local prevalence of food insecurity, which may inform the development of health care–based interventions to address this issue [8]. Additionally, our relatively small population of individuals with very low food security limits the generalizability of estimates for the performance of HVS for this particular subgroup. Finally, this study's use of zip codes to identify potentially eligible participants, due to the technical capacity of the EMR to extract geocodes for this study, may be a limitation; the use of zip codes, as opposed to census tracts, may mean that this study oversampled individuals who had higher socioeconomic status. However, the inclusion of higher income individuals is unlikely to have altered the performance of the HVS versus the HFSSM.

In summary, the HVS has high accuracy for detecting food insecurity in pregnant populations. The HVS may be a brief, useful tool to detect food insecurity in pregnancy if integrated into antenatal care.

## CONFLICT OF INTEREST STATEMENT

The authors declare no conflicts of interest.

## Data Availability

The data from this study are not publicly available but may be provided upon reasonable request to the authors.
